# Barrier-free tomato fruit selection and location based on optimized semantic segmentation and obstacle perception algorithm

**DOI:** 10.3389/fpls.2024.1460060

**Published:** 2024-10-31

**Authors:** Lingli Zhou, Anqi Hu, Yawen Cheng, Wenxiang Zhang, Bingyuan Zhang, Xinyu Lu, Qian Wu, Ni Ren

**Affiliations:** ^1^ Institute of Agricultural Information, Jiangsu Academy of Agricultural Sciences, Nanjing, China; ^2^ Key Laboratory of Intelligent Agricultural Technology (Changjiang Delta), Ministry of Agriculture and Rural Affairs, Nanjing, China

**Keywords:** harvesting robot, image semantic segmentation, obstacle perception, deep learning, fruit selection, positioning, tomato

## Abstract

With the advancement of computer vision technology, vision-based target perception has emerged as a predominant approach for harvesting robots to identify and locate fruits. However, little attention has been paid to the fact that fruits may be obscured by stems or other objects. In order to improve the vision detection ability of fruit harvesting robot, a fruit target selection and location approach considering obstacle perception was proposed. To enrich the dataset for tomato harvesting, synthetic data were generated by rendering a 3D simulated model of the tomato greenhouse environment, and automatically producing corresponding pixel-level semantic segmentation labels. An attention-based spatial-relationship feature extraction module (SFM) with lower computational complexity was designed to enhance the ability of semantic segmentation network DeepLab v3+ in accurately segmenting linear-structured obstructions such as stems and wires. An adaptive K-means clustering method was developed to distinguish individual instances of fruits. Furthermore, a barrier-free fruit selection algorithm that integrates information of obstacles and fruit instances was proposed to identify the closest and largest non-occluded fruit as the optimal picking target. The improved semantic segmentation network exhibited enhanced performance, achieving an accuracy of 96.75%. Notably, the Intersection-over-Union (*IoU*) of wire and stem classes was improved by 5.0% and 2.3%, respectively. Our target selection method demonstrated accurate identification of obstacle types (96.15%) and effectively excluding fruits obstructed by strongly resistant objects (86.67%). Compared to the fruit detection method without visual obstacle avoidance (Yolo v5), our approach exhibited an 18.9% increase in selection precision and a 1.3% reduction in location error. The improved semantic segmentation algorithm significantly increased the segmentation accuracy of linear-structured obstacles, and the obstacle perception algorithm effectively avoided occluded fruits. The proposed method demonstrated an appreciable ability in precisely selecting and locating barrier-free fruits within non-structural environments, especially avoiding fruits obscured by stems or wires. This approach provides a more reliable and practical solution for fruit selection and localization for harvesting robots, while also being applicable to other fruits and vegetables such as sweet peppers and kiwis.

## 1 Introduction

Fruit picking is a time-consuming and laborious work, accounting for 33%-50% of the total production workload. With the advancement of agricultural modernization, the research on harvesting robots has attracted much attention. Harvesting robots will be the key technology to address the issue of labor shortage in the future. This advancement encompasses multidisciplinary techniques spanning kinematics, control science, machine vision, and behavioral science. Following several years of development, substantial progress has been made in addressing key technical challenges such as path planning (Ye et al., 2023; Ghosh et al., 2019; Tang et al., 2024), systematic control (Chen et al., 2024), target recognition and positioning (Guo et al., 2023; Yang et al., 2023), and picking sequence planning (Kurtser and Edan, 2020). Nowadays, various prototypes of single-fruit harvesting robots have been developed, such as tomato harvesting robots (Jun et al., 2021), apple harvesting robots (Zhang et al., 2021; Silwal et al., 2017), kiwifruit harvesting robots (Williams et al., 2019) and so on. The visual servo harvesting robot (Williams et al., 2019; Miao et al., 2023; Jiao et al., 2022) stand out as one of the research hotspots due to their significant advantages in autonomy, precision, and adaptability. The first and most crucial step for these camera-based robots is to detect and locate the target to be harvested in the visual image, and obtain the position of the target in three-dimensional (3D) through coordinate transformation with the help of depth maps or other auxiliary information. Despite significant breakthroughs (Montoya-Cavero et al., 2022; Rong et al., 2022) have been made in fruit recognition and positioning based on visual images, the uncertainty in fruit growth and the complexity of unstructured orchard environments still lead to many problems for the visual system of harvesting robots.

Before deep learning was widely used, traditional machine learning algorithms combined with image processing were the most common methods for fruit recognition. Through a series of pre-processing, such as color space transformation, image denoising, edge detection and region growth (Ouyang et al., 2013; Wachs et al., 2010; Zhao et al., 2016), different levels of features were extracted and served as the input of the machine learning methods for image classification. However, the traditional machine learning algorithms are difficult to deal with images collected in complex natural environments, and their accuracy are affected by light intensity and ray shadow, so their robustness are not enough to meet practical needs.

Compared to traditional machine learning, deep learning characterized by its superior representation, learning, and generalization abilities, has garnered significant attention and widespread application. The deep learning-based computer vison technology has significantly advanced the development of harvesting robots and accelerated their practical application. The most commonly used methods (Divyanth et al., 2022; Kuznetsova et al., 2020; Yan et al., 2021) for harvesting robots are to customize position calculation algorithms for specific species by improving the object detection models, including Faster Regional-Convolutional Neural Network (Faster R-CNN) (Ren et al., 2016) and YOU LOOK ONLY ONCE (YOLO) (Redmon et al., 2016). In order to achieve the real-time detection of apples or oranges, Kuznetsova et al (Kuznetsova et al., 2020). designed the pre- and post-processing techniques based on YOLO v3 algorithm, which shortened the average detection time to 19 ms. Although the algorithms based on object detection have achieved promising results in terms of speed, they can only estimate the location and size of the target. For some soft-rind fruits harvested by cutting the stems to prevent damage to the epidermis, more appearance information, such as the contour of targets, is required to determine the positions of the cutting points. The image semantic segmentation and instance segmentation provide good methods to meet this kind of harvesting demand. The work presented by Yu et al (Yu et al., 2019). was a typical case of applying the instance model Mask Region Convolutional Neural Network (Mask R-CNN) (He et al., 2020) to strawberry harvesting, which calculated picking points on mask images generated by Mask R-CNN. In order to recognize and segment overlapped apples, Jia et al (Jia et al., 2020). optimized Mask R-CNN by combining Residual Network (ResNet) (He et al., 2016) with Densely Connected Convolutional Networks (DenseNet) as an alternative to its original backbone network for reducing input parameters, which was ideally effective in terms of both speed and accuracy of target positioning tested on a random test set. It is a trend to use semantic segmentation (Li et al., 2021; Kang and Chen, 2020) and instance segmentation (Luo et al., 2022; Zheng et al., 2021) to solve target recognition and location in the vision system of harvesting robots, as it can provide more appearance information and realize the separation of fruits and background, and individual targets.

Although these methods have achieved notable breakthroughs, they still face difficulties in addressing the challenges presented by complex growth environments. We have noticed that the tomato fruits grown under natural conditions are often obscured by various objects such as leaves, stems, wires and other fruits. The presence obstacles, particularly wires and stems, poses a significant challenge to mechanical harvesting by impeding the movement of robotic arms. The entanglement between these slender objects with strong resistance and the robotic arm may lead to fatal errors, so the issue of obstacle occlusion has gradually attracted the attention of researchers. Divyanth et al (Divyanth et al., 2022). has improved the Faster R-CNN model by adding an attention mechanism to detect non-occluded coconuts and leaf-occluded coconuts. However, this method cannot meet the perceptual need for more intricate scenarios. The information of the obstacles, including stems, wires, branches and petioles, need to be captured in semantic level because they directly affect the selection of picking targets and the path planning of picking execution agencies. In recent years, semantic segmentation algorithms have been employed to segment objects with linear structure (such as stems, branches, etc.) in unstructured environments. Song et al. (Song et al., 2021) proposed a branches and wires segmentation and reconstruction method for kiwifruit. Wang et al. (Wang et al., 2023) proposed a parallel network structure (DualSeg) to segment branches and fruits for grapes. Although these studies have successfully implemented pixel-level perception of obstacles through image semantic segmentation, their approach lacks the integration of both obstacle perception and fruit instance discrimination, as well as an analysis of fruit occlusion in the orchard environment.

Thus, we proposed our method to locate fruit instances while recognizing obstacles based on the following two considerations. Firstly, in order to distinguish fruits from different obstacles, especially linear-structured obstacles, an improved image semantic segmentation algorithm was proposed. Due to the slender structure of major obstacles such as stems and wires, we added spatial-relationship features to the semantic network, which was inspired by the research (Pan et al., 2018; Gioi et al., 2012) used to identify slender objects such as lane lines and transmission lines. Different from the slice-by-slice convolution used in Spatial Convolutional Neural Network (SCNN) (Pan et al., 2018), we redesigned an attention-based spatial-relationship feature extraction module with lower computational complexity to enhance the ability of semantic segmentation network to recognize obstacles with strong shape priors. The module skillfully applied spatial attention masks to transmit information in the rows and columns of features, thereby changing the attention allocation of network and achieving more attention to slender structural objects to improve their segmentation accuracy. Secondly, an adaptive K-means (MacQueen, 1967) pixel clustering algorithm was designed to segment fruit instances based on the characteristic of different fruit depths and positions, effectively addressing the challenges associated with unsatisfactory clustering performance due to a fixed K value and the uncertain number of fruits in each image. Subsequently, a straightforward yet efficient barrier-free fruit selection algorithm combining the information of obstacles and fruit instances was proposed to select the closest and largest non-occluded fruit as the ultimately picking target. The feasibility of our method was validated on our Tomato dataset, which performed well in both semantic segmentation, and selection and location of barrier-free fruits.

## 2 Materials and methods

### 2.1 Tomato dataset

#### 2.1.1 Image acquisition

The images were acquired at the Tomato Intelligent Production Greenhouse of Jiangsu Academy of Agricultural Sciences, Nanjing, China (32.03° N, 118.87° E) in 2022-2023. All tomatoes were grown using soilless culture, and two tomato cultivars “Sufen No.6” and “Fatalong” were selected for data acquisition.

**Figure 1:**
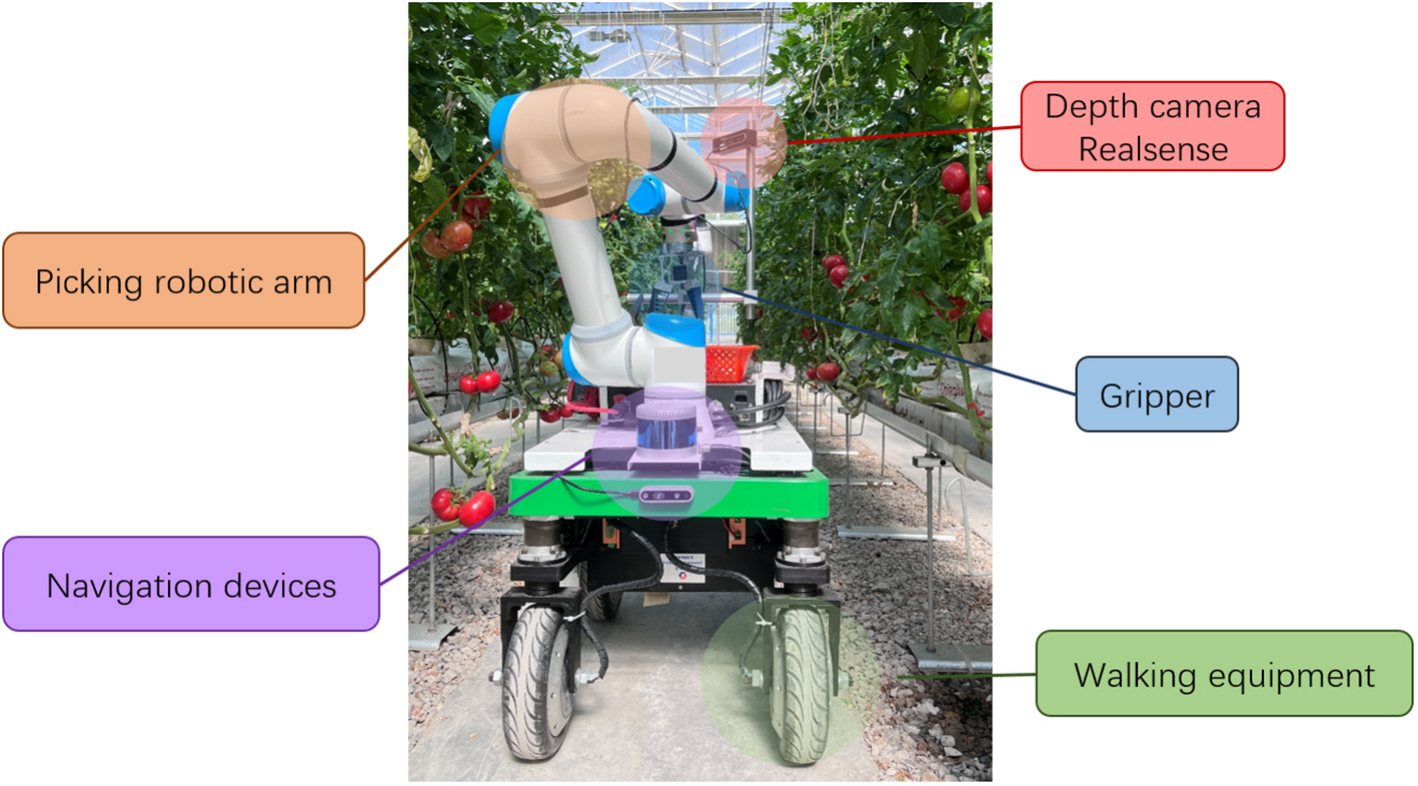
The developed tomato harvesting robot.

A tomato harvesting robot platform composed of a picking robotic arm, a visual system, a control system and a walking equipment was developed (**Figure 1**), and the intelligent picking operations, such as picking path planning, picking target identification and positioning, and non-destructive picking, were realized. A depth camera Realsense (D435i, Intel, America) mounted on the tomato harvesting robot was used to automatically capture RGB images and depth images with a resolution of 600×800 pixels. The tomato harvesting robot stopped every 1 meter for tomato data collection. The images were taken irregularly between 9 a.m. and 5 p.m. under different weather conditions (sunny and cloudy) from February to April. Images that were too bright, too dark or in other colors that do not meet the standards were removed, and finally 170 RGB images and corresponding depth maps were selected.

#### 2.1.2 Image annotation and dataset production

Due to the limited number of empirical images, we used the synthetic data generated by the method provided by Barth et al. (Barth et al., 2018) to pre-train the weights of the semantic segmentation model. 3250 synthetic tomato images and corresponding pixel-level synthetic semantic segmentation label images were generated by rendering on the 3D simulated model of tomato greenhouse environment produced by Blender. The resolution of all synthetic images was 1600×1200 pixels, and each pixel in the synthetic images corresponds to one of nine classes (background, mature fruits, immature fruits, peduncles, stems, branches and petioles, wires, cuts, leaves) represented by a color in the label images.

170 empirical RGB images were labelled with pixel-level semantic segmentation using the open source software Labelme (Russell et al., 2008), with 120 images as training data, 20 images as validation data, and the remaining 30 images as testing data. The annotators annotated the images from back to front, ensuring that the boundary of each object was not marked repeatedly. Considering the labelling speed and quality, only the first row of plants in the images were labelled, and other distant plants can be regarded as background because they have little effect on the perspective of robots. We omitted the annotation of leaves for empirical images, mainly based on the following two considerations: (i) the leaves were regularly removed during the harvesting seasons, and the presence of a limited number of leaves does not significantly impact the operation of the robots; (ii) the morphology of tomato leaves was complex and irregular, resulting in a significant increase in the workload associated with data annotation. Therefore, there were a total of 8 label classes (background, mature fruits, immature fruits, peduncles, stems, branches and petioles, wires, cuts) in empirical label images and the annotation time for each image took about 1 hour.

**Figure 2:**
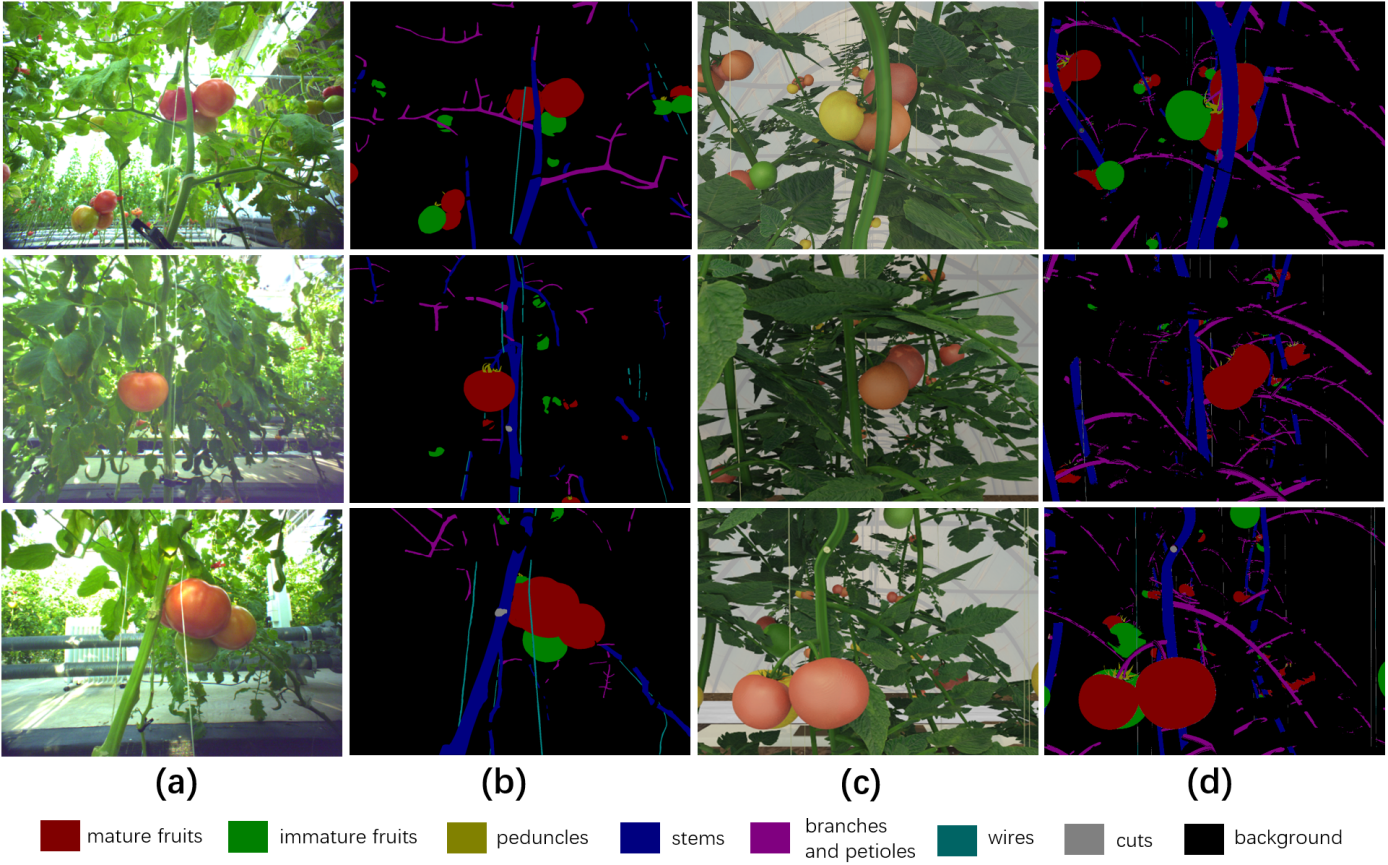
Visual comparison between synthetic data and empirical data. **(A)** Three empirical images, **(B)** corresponding semantic segmentation labels of three empirical images, **(C)** three synthetic images, **(D)** corresponding synthetic labels of three synthetic images. For better comparison, the leaves in the synthetic labels are displayed as background.

**Figure 2** shows some examples of empirical data and synthetic data. It can be seen that the synthetic images and empirical images have high similarity, and fruits in both are easily obscured by objects such as wires and stems.

### 2.2 Proposed method for barrier-free fruit selection and location

**Figure 3:**
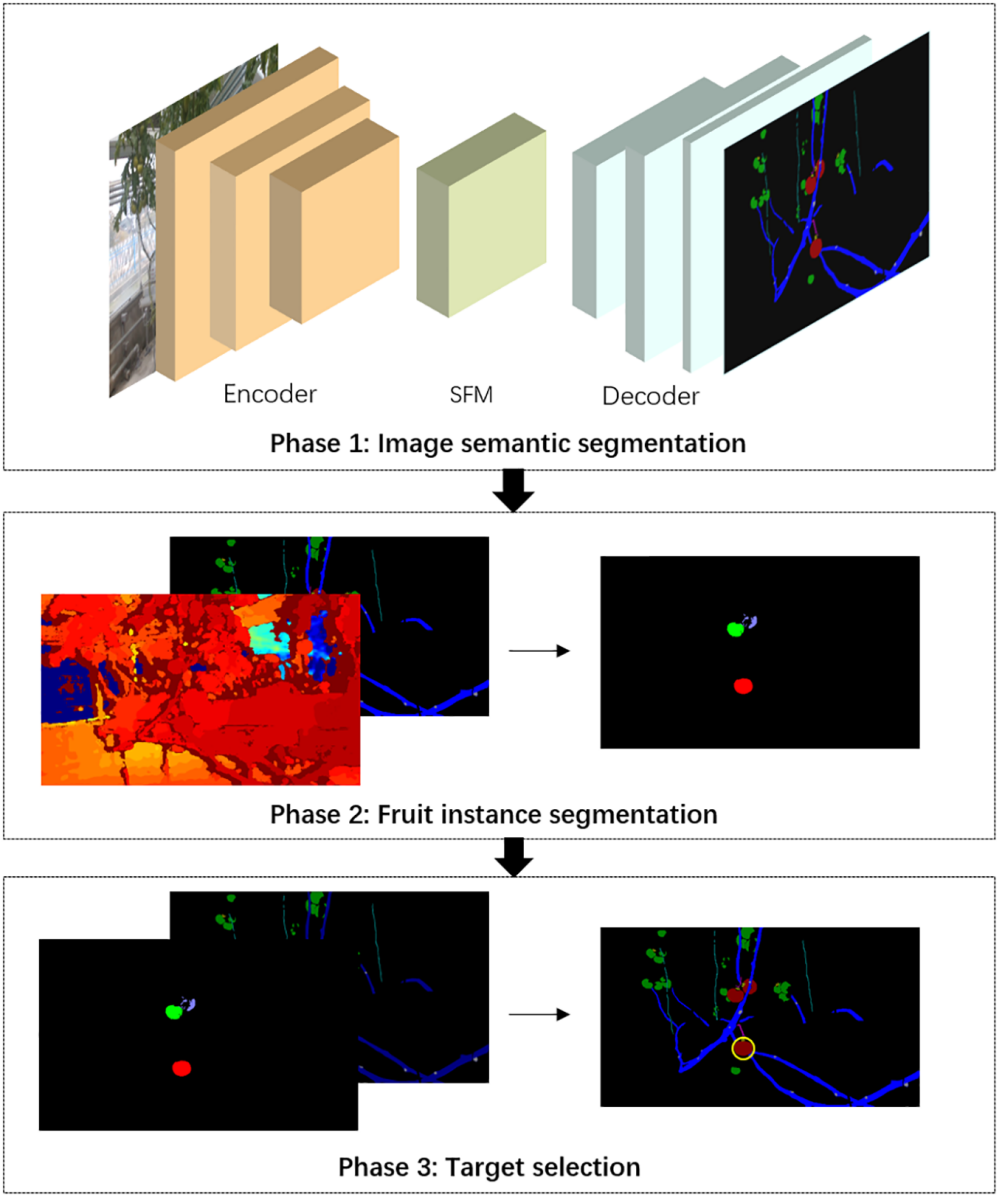
The flowchart of the non-occluded fruit selection and location approach for single-fruit harvesting robots.

In order to solve the problem of barrier-free fruit selection and positioning based on monocular images, a segmentation and location approach for vision system of harvesting robots based on RGB images and depth maps was proposed. The whole flowchart was divided into three phases (**Figure 3**). In phase 1, an improved semantic segmentation network was used to distinguish fruits, obstacles and background. The shape and location of the major obstacles, such as stems, branches and petioles, wires, need to be delicately identified. Therefore, we improved the recognition accuracy of obstacles with slender structures by adding spatial-relationship feature module (SFM) into the encoder of DeepLab v3+ (Chen et al., 2017), and achieved high-precision pixel-level segmentation of plant organs and key objects. In phase 2, based on the position information of fruits in the semantic segmentation maps and the depth information provided by depth maps, an adaptive K-means clustering method was applied to cluster pixels of fruits into individual instances. In phase 3, barrier-free harvesting targets were selected by using a proposed obstacle detection method, and the optimal picking target was recommended.

#### 2.2.1 Improved image semantic segmentation network

The widely used DeepLab v3+ (Chen et al., 2017) was adopted as the baseline of the image semantic segmentation network, and its encoder was optimized to enhance the segmentation ability of slender obstacles. Inspired by SCNN for lane detection (Pan et al., 2018), we proposed a spatial-relationship feature module (SFM) and strengthened the spatial relationships of pixels across rows and columns for objects with strong shape priors but weak appearance coherences during feature extraction.

Given the demand for real-time inference in agricultural machinery automation, it is imperative to develop accurate, lightweight, and fast models. Considering the computational complexity of SCNN, we proposed a lighter spatial information perception module drawing on the idea of the spatial attention mechanism (Jaderberg et al., 2015). Generally speaking, the spatial attention mechanism assigns different weights to pixels through a mask, thereby directing more attention towards regions of interest. In our study, it is essential to increase space-attention towards stems and wires to enhance their segmentation accuracy. These objects exhibit obvious directionality in both horizontal and vertical directions in the images. In light of these considerations, we proposed our spatial-relationship feature module (SFM). This module was designed to augment the spatial attention mechanism by incorporating the inherent directional attributes of the target objects, thereby enhancing the segmentation accuracy.

**Figure 4:**
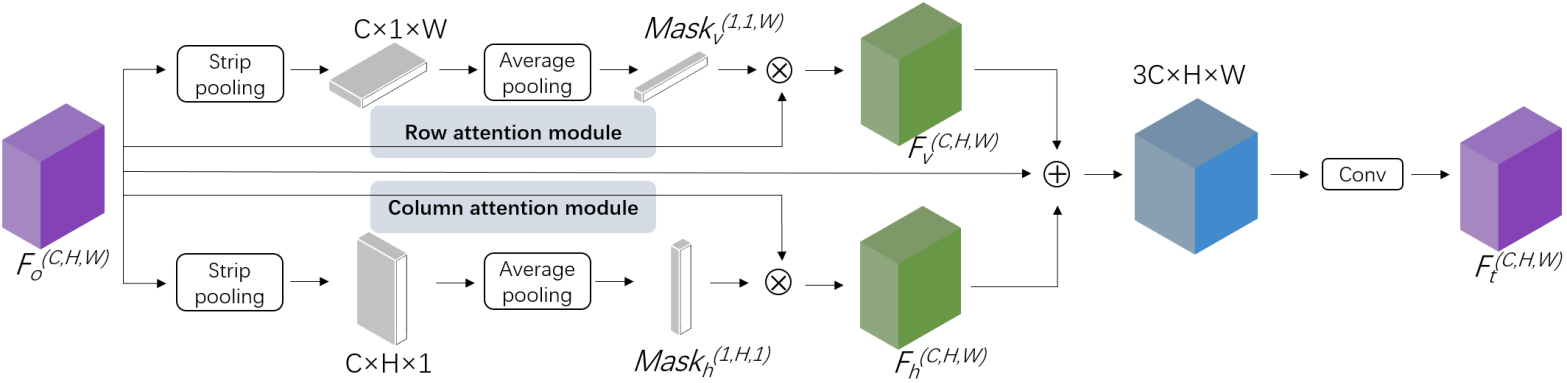
Sketch of spatial-relationship feature module (SFM) of our approach.


SFM presented a novel methodology for feature transformation that differs from the traditional slice-by-slice convolution utilized by SCNN. SCNN operated in four computational directions: top to bottom, bottom to top, right to left, and left to right. In each direction, the features were sliced, with individual convolutions applied to each slice and addition operations conducted between slices. In contrast, SFM utilized two masks derived from stripe pooling of the original features along their spatial directions to facilitate information transmission across the rows and columns of features. This innovative approach significantly reduced the parameter count, thereby enhancing model efficiency. As the masks were derived from the features, the spatial information of the original features was retained. Subsequently, the two masks were dot-multiplied with the original features in their respective responsible directions, thereby reallocating attention to the original features. This process effectively reallocated the focus within the original features, facilitating a more nuanced and context-aware representation of features. As shown in **Figure 4**, letting *F_o_^(C,H,W)^* denote the 3-D features generated from the feature extraction network ResNet101 of DeepLab v3+ for an image. The extraction of spatial features was conducted through a dual-axis approach, focusing on both the horizontal and vertical orientations within the image. Horizontal stripe pooling with the kernel size of 1×N compressed *F_o_^(C,H,W)^* to the size of C×H×1, followed by an average pooling of the channels to obtain smaller features with a size of 1×H×1. Through the nonlinear activation function ReLU, the attention mask *Mask_h_^(1,H,1)^* on the horizontal dimension was calculated, as described in Equation 1. Then, the mask was dot multiplied by the original feature *F_o_^(C,H,W)^* to capture the horizontal dependencies and obtain the horizontal spatial relationship feature *F_h_^(C,H,W)^*, as seen in Equation 2. Similarly, the vertical spatial relationship feature *F_v_^(C,H,W)^* was calculated. *F_o_^(C,H,W)^*, *F_v_^(C,H,W)^* and *F_h_^(C,H,W)^* was merged by cascading, and then the output was fed into the convolution layer to generate the final features *F_t_^(C,H,W)^* with spatial relationships, as seen in Equation 3.

1 $$Mask_{h}^{\left(1,H,1\right)}=ReLU\left(AvgPool\left(StripPool\left(F_{o}^{\left(C,H,W\right)}\right)\right)\right)$$

2 $$F_{h}^{\left(C,H,W\right)}=F_{h}^{\left(C,H,W\right)}\times Mask_{h}^{\left(1,H,1\right)}$$

3 $$F_{t}^{\left(C,H,W\right)}=Conv\left(Concat\left(F_{o}^{\left(C,H,W\right)},F_{h}^{\left(C,H,W\right)},F_{v}^{\left(C,H,W\right)}\right)\right)$$

where *C*, *H*, *W* stand for the number of channels, rows, and columns respectively, *ReLU* denotes nonlinear activation function, *AvgPool* denotes average pooling, *StripPool* denotes strip pooling, × denotes dot multiplication operation, *Conv* denotes standard convolution and *Concat* denotes cascade operation.

This specially designed module facilitated the transmission of pixel feature information within the layer space and enabled the network pay more attention to the objects with slender structures in both rows and columns. The attention masks were obtained from spatial feature statistics with specific directionality, which help original features perceive information in the row and column space of the layer. Specially, the SFM module has low computational complexity. Taking a feature of size 1×64×64×128 as an example, the computational cost of performing one SCNN operation on it exceeds 75.5 MFLOPs, whereas the computational cost for SFM is approximately 2.6 MFLOPs. Meanwhile, the design of SFM ensures compatibility with existing feature extraction frameworks, allowing for seamless integration following the feature extraction backbone of the segmentation network.

#### 2.2.2 Segmentation for fruit instance

In general, pixels belonging to the same instance have close positions in semantic segmentation maps and similar depth values in depth maps, even if they were partitioned by obstacles. A clustering method based on K-means (MacQueen, 1967) was chosen to divide the pixels of mature fruits into different instances with the help of depth information.

The clustering algorithms could represent a more efficient method in contexts with constrained computational resources. K-means clustering is a straightforward and efficient unsupervised learning method that offers the advantage of applicability to various types of data, thereby making it widely utilized in image segmentation tasks (Zhang and Peng, 2022). The basic idea of the algorithm is to find the optimal partition scheme of K-means clustering by minimizing the loss function. The loss function is the sum of the distances from all the elements to the center of the cluster, we defined our loss function *L* in Equation 4,


4 $$L=\sum_{j=1}^{\text{k}}\sum_{i=1}^{N_{j}}{\left\|p\left(X_{\text{i}}\right)-p\left(Z_{i}\right)\right\|}^{2}+\alpha {\left\|d\left(X_{\text{i}}\right)-d\left(Z_{\text{j}}\right)\right\|}^{2},X_{\text{i}}\in S_{j}$$

where, k is the number of clusters, *S_j_* is the cluster set, and *N_j_* and *Z_j_* are the number of pixels and the cluster center belonging to *S_j_* respectively, *p(x)* denotes the position of *x* and *d(x)* denotes the depth value of *x*, ||·||^2^ is the *L2*-norm, *α* is used to balance the proportion of two parts.

The clustering centers were continuously optimized through many iterations until they no longer changes. The resolution of the original images needs to be reduced before pixel clustering in order to reduce the noise and improve the operational efficiency. The initial clustering centers were randomly selected from the established mature fruit categories to improve clustering efficiency. Considering the different number of fruits in different images, we adopted an adaptive K-means clustering algorithm for each image. Specifically, we initially selected a larger value as the original K-value based on the count of mature tomatoes in the foreground of the images from previous trials. After clustering on this K-value, the results were checked and the closest clustering centers were merged. Then, the K-value was updated, and the next round of clustering continued until no new K-value was generated.

#### 2.2.3 Target selection and location

**Figure 5:**
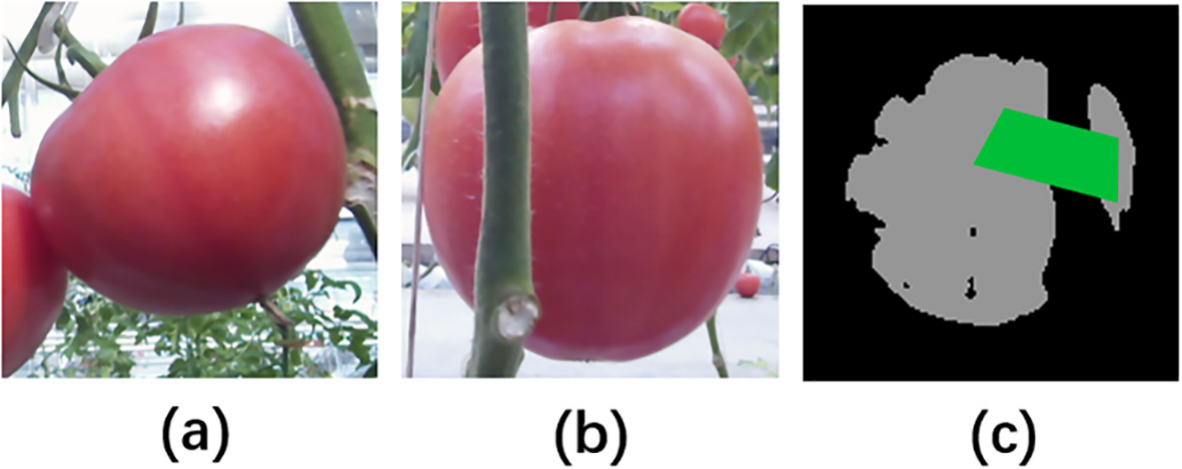
Tomato fruits with different regional connectivity and fruit blind spot identification. **(A)** A fruit without any obstruction, **(B)** a fruit occluded by a stem, **(C)** a blind spot generated by two connected regions represented by a green region.

A target selection method was proposed to screen the fruit instances obtained in the second phase based on the obstruction information obtained in the first phase. The barrier-free instances were retained and prioritized based on their area and depth values. Ideally, a picking instance would be a connected region (**Figure 5A**), while the instances occluded by objects (such as stems) would be divided into multiple regions (**Figure 5B**). Therefore, we first used a basic connected region analysis algorithm (Di Stefano and Bulgarelli, 1999) to label connected regions of the binary image mask generated from each instance. Then, we randomly selected any two points from two non-connected regions, and connected the four points into a quadrilateral, which was defined as a blind spot, as shown in the green area in **Figure 5C**. If an object divided an instance into two regions, the object was likely to exist in the corresponding blind spot region on the semantic segmentation map. Therefore, all blind spots were sent to semantic segmentation maps to check for the presence of obstacles.

By correlating the blind spots with the semantic segmentation map, the corresponding categories of obstacles, including stems, wires, branches and petioles, were identified. In the absence of a corresponding category within the blind spots, which signified an empty background, the obstacles were initially classified as leaves. This assumption was primarily based on the observation that only leaves or other obstacles could partition a single fruit instance into multiple regions, thereby creating blind spots; conversely, the background itself could not divide fruit instances and generate these blind spots. To enhance the rigor of obstacle detection, we employed the Otsu threshold segmentation algorithm (Yousefi, 2011) based on color features to determine whether the obstacles in the blind spots belong to leaves or unknown categories. Specifically, the Otsu algorithm performed rapid binary segmentation on the remaining portion of the original image identified as background, effectively distinguishing leaves from other elements within that background based on their distinct color features. Subsequently, the obstacles obstructing the fruit were further clarified by referencing the binary segmentation result of the non-fruit area in the blind spots.

When an instance presented obstacles (stems, branches and petioles, wires) in its blind spots, it would be discarded. If an instance was obstructed by leaves, it would still be retained as the obstruction caused by leaves does not affect the mechanical picking. The center coordinates and radii of the remaining instances were obtained by delineating the minimum circumscribed circles of the connected regions. Then, the closest and largest non-occluded fruit was selected as the ultimate harvesting target according to the comprehensive evaluation scores calculated from their areas and depth values. The algorithm flow was shown in **Supplementary Material 1**.

### 2.3 Experiments and performance evaluation

#### 2.3.1 The optimized algorithm enhances the performance of image semantic segmentation

The method presented above was tested on the on our Tomato dataset. The whole approach was implemented in the Python programming language. For image semantic segmentation, deep CNNs was implemented on TensorFlow (Cordts et al., 2016), a public deep learning architecture. Adam optimizer (Kingma and Ba, 2014), which is computationally efficient and widely used in many models, was used to improve the performance of the networks. Meanwhile, the “exponential decay” policy was used to control the updating speed of parameters and accelerate the convergence speed of the networks. During the whole training process, original input was randomly cropped. The crop size was 640×640 pixels and batch size was 4. Some layers were added a “dropout” strategy to prevent over-fitting during the training process. The networks were trained with the pre-trained parameters from ImageNet (Russakovsky et al., 2015), and each model converged after approximately 60k iterations. For fruit instances segmentation and the post-processing including the selection of picking targets, their implementation relied on OpenCV, an open-source library for computer vision.

#### 2.3.2 The evaluation of image semantic segmentation

The image semantic segmentation performance of DeepLab v3+ and DeepLab v3+ with SFM were compared. According to the conventional evaluation criteria, Pixel Accuracy (*acc*) and Intersection-over-Union (*IoU*) (Everingham et al., 2015) were adopted as evaluation criteria for image semantic segmentation. The *acc* was defined as the ratio of all correctly classified pixels to all valid pixels in the image (Equation 5). The *IoU* evaluated the similarity between the portion parsed by the network and ground truth related to a specific class (Equation 6).


5 $$acc=\frac{1}{N}\sum_{i=1}^{N}\frac{R_{i}}{V_{i}}\times 100\%$$

6 $$IoU=\frac{1}{N}\sum_{i=1}^{N}\frac{P_{i}\cap G_{i}}{P_{i}\cup G_{i}}\times 100\%$$

where *N* is the number of images in the testing set; *R_i_* and *V_i_* are the total number of correctly classified pixels and the total number of valid pixels in image *i*, respectively; *P_i_* denotes the area predicted as the target class, *G_i_* represents the area of the target class in the ground truth. Therefore, the value of *IoU* was calculated by dividing the intersection area of the two regions by the union area of two regions. *IoU* was a supplement to *acc*, it took into account the class imbalance that existed in most datasets. In addition, the mean intersection-over-union (*mIoU*) of all classes was added as another indicator.

#### 2.3.3 The evaluation of fruit instance segmentation

The effectiveness of the proposed adaptive K-means clustering algorithm was validated by evaluating the fruit instance segmentation performance using different K values. Generally, the number of mature fruits in each image’s foreground should not exceed 8, hence we varied the K value within a range from 2 to 8. Precision, Recall and F1 were employed as evaluation metrics for fruit instance segmentation performance assessment. Usually, Precision and Recall are contradictory, with high Precision resulting in low Recall, and vice versa. Therefore, F1 Score was often used to comprehensively measure the performance of a model while balancing Precision and Recall. The three indicators were defined in Equations 7–9,


7 $$Precision=\frac{N_{TP}}{N_{TP}+N_{FP}}\times 100\%$$

8 $$Recall=\frac{N_{TP}}{N_{TP}+N_{FN}}\times 100\%$$

9 $$F1=\frac{2\times Precision\times Recall}{Precision+Recall}\times 100\%$$

where *N_TP_* is the number of positive samples correctly identified as positive, *N_FP_* is the number of negative samples incorrectly identified as positive, and *N_FN_* is the number of positive samples incorrectly identified as negative. If the ratio of the correctly detected area to the ground truth exceeds a threshold, the target was considered to be correctly detected.

For the evaluation of fruit instance segmentation performance, the threshold of *IoU* was set to 0.5, indicating that if the overlapping area between the segmented fruit instance and manually labelled instance exceeded 50% of the latter, it would be marked as a true positive. Normally, *IoU* values exceeding 0.5 could support the harvesting robot for picking operations.

#### 2.3.4 The evaluation of target selection and location approach

The performance of the approach in picking-targets selection was evaluated from two aspects of the target selection and the location precision. *Precision*, *Recall* and *F1* were commonly used metrics for target detection. The central position and radius of the fruit were the key parameters for the harvesting robots to perceive the target position. The positioning error (*PE*) for a target was defined in Equation 10,


10 $$PE=\frac{{\left\|C_{p}-C_{g}\right\|}^{2}}{R}\times 100\%=\frac{\sqrt{{\left(x_{p}-x_{g}\right)}^{2}+\left(y_{p}-y_{g}\right)}}{R}\times 100\%$$

where *C_p_* and *C_g_* and are the center coordinates predicted by the algorithm and the manually labeled center, respectively, and *R* is the target radius. *PE* was essentially a relative error between positions.

## 3 Results

### 3.1 The diversity and robustness of the empirical data

The empirical images were collected at various temporal intervals and under diverse lighting conditions to cope with different harvesting situations. Different weather conditions have a significant impact on the quality of image acquisition and subsequent image processing tasks. Under overcast conditions, inadequate illumination may produce excessively dark images, resulting in a loss of detail, whereas bright light can lead to overexposure. Collecting images under different weather conditions can effectively enhance the richness and robustness of the image dataset, thereby providing substantial value in improving the model’s applicability across different weather conditions.

To assess the effectiveness, diversity and robustness of our empirical data, we created two training datasets by dividing the original dataset into two categories based on the weather conditions. Then, we evaluated the semantic segmentation performance of DeepLab v3+ trained on different datasets, including images captured under diverse weather conditions, as well as those taken in sunny and cloudy weather conditions (**Table 1**). All three models were pre-trained on the synthetic data and the training images were augmented through geometric transformation and color transformation. The results indicated that the model trained on the dataset comprising images captured under diverse weather conditions significantly outperformed the other two models in terms of accuracy (*acc*) and mean Intersection over Union (*mIoU*), as both of their training datasets exhibited notable biases. This finding demonstrated the diversity and robustness of our empirical training data.

**Table 1:** The accuracy (*acc*) and mean Intersection over Union (*mIoU*) of DeepLab v3+ trained on different datasets.

Training data type	*acc* (%)	*mIoU* (%)
**Images taken under diverse weather conditions**	96.53	62.12
**Images taken under sunny weather conditions**	92.00	45.40
**Images taken under cloudy weather conditions**	92.41	45.27

### 3.2 The optimized algorithm enhances the performance of image semantic segmentation

**Figure 6:**
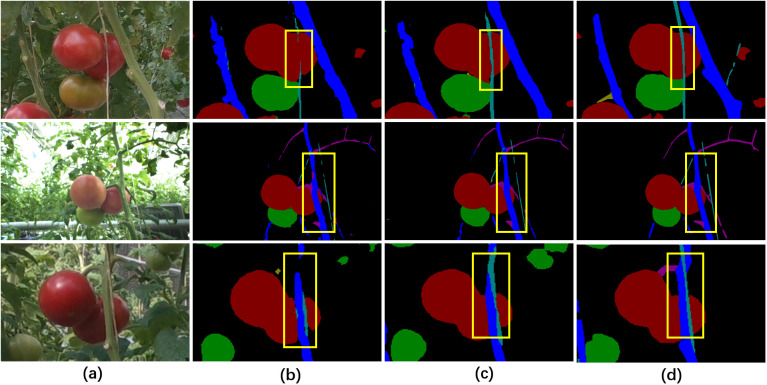
Visual comparison of the parsing results of different semantic segmentation methods. **(A)** Original images, **(B)** the segmentation results of DeepLab v3+, **(C)** the segmentation results of DeepLab v3+ with SFM, **(D)** the ground truth labels. Class labels: black indicates background, red indicates mature fruits, green indicates immature fruits, yellow indicates peduncles, dark blue indicates stems, purple indicates branches and petioles, light blue indicates wires and gray indicates cuts.

The performance of image semantic segmentation has a significant impact on the subsequent single-fruit segmentation, obstacles perception and target selection. The performance of the widely used semantic segmentation network DeepLab v3+ and the proposed DeepLab v3+ with SFM were compared on the Tomato dataset. The visual comparison results showed that the segmentation of wires and stems were significantly enhanced by using DeepLab v3+ with SFM (**Figure 6**), demonstrating that SFM can effectively assist semantic segmentation networks to parse objects with slender structures more accurately.

In the quantitative comparison, we included U-net network (Ronneberger et al., 2015) as an additional baseline network and compared it with the model with SFM. The quantitative analysis results showed that both models with SFM achieved better performance, while DeepLab v3+ with SFM exhibited the best semantic segmentation performance (**Table 2**). The *acc* of DeepLab v3+ with SFM reached 96.75% and the *mIoU* exceeded that of DeepLab v3+ by a gap of 2.19%. The *IoU* values of most classes have been improved to varying degrees due to the introduction of spatial relationship through SFM (**Table 3**), especially for obstructions with slender structures. Notably, significant enhancements were observed in the classes of stems and wires (**Table 3**), where U-net with SFM showed a respective increase of 2.48% and 5.07% and DeepLab v3+ with SFM exhibited an increase of 2.30% and 5.00%. Overall, the optimized approach resulted in more accurate and smooth segmentation due to the spatial-relationship perception ability of SFM, which was beneficial to the recognition of obstructed fruits.

**Table 2:** The accuracy (*acc*) and mean Intersection over Union (*mIoU*) of different semantic segmentation methods.

Method	*acc* (%)	*mIoU* (%)
**U-net**	91.74	50.28
**U-net with SFM**	92.94	52.81
**DeepLab v3+**	96.53	62.12
**DeepLab v3+ with SFM**	96.75	64.31

**Table 3:** The *IoU* of different semantic segmentation methods for different classes.

Method	Background (%)	Mature fruits (%)	Immature fruits (%)	Stems (%)	Wires (%)	Branches and petioles (%)
**U-net**	92.28	81.36	60.98	51.59	31.18	36.22
**U-net with SFM**	92.87	83.10	68.43	54.07	36.25	35.69
**DeepLab v3+**	96.60	90.01	84.62	72.71	44.02	42.15
**DeepLab v3+ with SFM**	97.01	90.88	84.82	75.01	49.02	42.56

Moreover, the improvement of semantic segmentation performance by introducing SFM was also validated on a Pepper dataset (Barth et al., 2018) (**Supplementary Material 2**). The *acc* of DeepLab v3+ with SFM reached 90.56% and the *mIoU* exceeded that of DeepLab v3+ by a gap of 4.93% (**Supplementary Table S1**). Notably, significant enhancements were observed in the classes of stems, branches and leaf stems, and wires (**Supplementary Table S2**), where DeepLab v3+ with SFM exhibited an increase of 9.74%, 4.49% and 11.47% in *IoU* values. The result indicated that our algorithm exhibited strong generalization ability.

### 3.3 Positive effect of depth maps and adaptive K-means clustering on fruit instance segmentation

**Figure 7:**
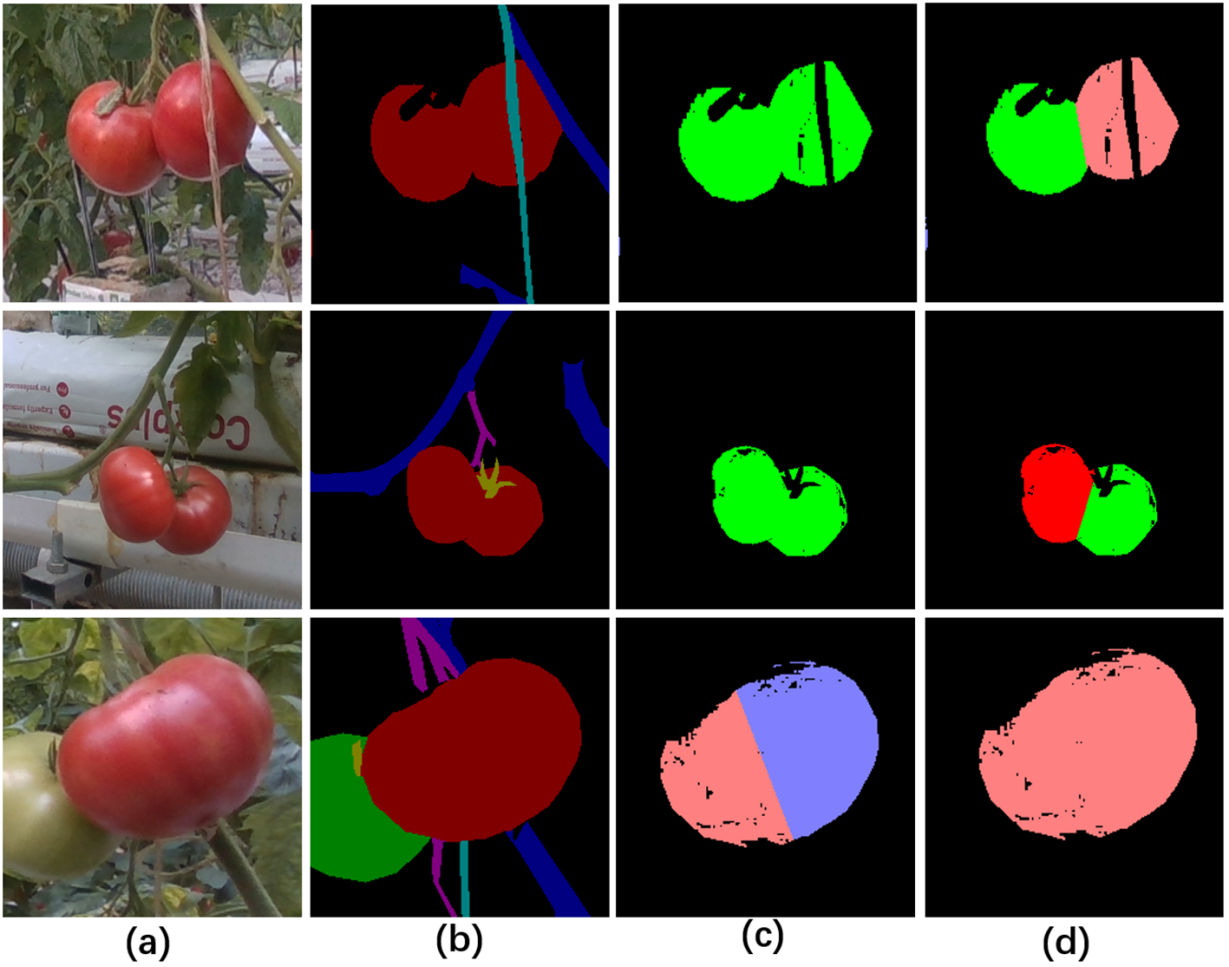
Pixel clustering with or without the information of depth maps. **(A)** Original RGB images, **(B)** image semantic segmentation for fruit differentiation, **(C)** visualization of pixel clustering of mature fruits without the information of depth maps, **(D)** visualization of pixel clustering of mature fruits with the information of depth maps.

The depth maps were used to assist in the segmentation of fruit instances in our method. The positive effect of depth maps on pixel clustering of fruit instances was shown in **Figure 7**, where the combination of depth maps and 2D images enabled the differentiation of adjacent fruits in 2D images based on different depth values (the first and second rows in **Figure 7**), while similar depth values also prevented oversized fruits from being cut apart (the third row in **Figure 7**). The reason for the preferable performance of instance segmentation was that depth maps provided more discriminating information.

The quantitative comparison results further proved the positive role of depth maps in segmentation of mature fruit instances (**Table 4**). When the depth maps were used as input of pixel clustering, the statistical results of fruit instance segmentation were significantly higher where the *Precision*, *Recall* and *F1* were improved by 4.8%, 12.8% and 9.44%, respectively.

**Table 4:** The performance of instance segmentation of mature fruits with and without the depth maps as input.

Method	*Precision* (%)	*Recall* (%)	*F1* (%)
**without depth maps**	87.20	70.83	78.17
**with depth maps**	92.00	83.63	87.61

Different K values were used to demonstrate the effectiveness of the proposed adaptive K-means clustering algorithm (**Table 5**). The statistical results showed that the adaptive K-means clustering had obvious advantages over different K-values in fruit instance segmentation. Although the Precision reached 94.11% when K was set to 2, both *Recall* and *F1* score were unsatisfactory (**Table 5**). When k was set to 4, *Precision*, *Recall* and *F1* reached 87.80%, 72.00% and 79.11% respectively, however, they still fell short of the adaptive K-means clustering method by 4.2%, 11.63% and 8.5%.

**Table 5:** Comparison of instance segmentation performance of mature tomato fruits under different K values.

K	*Precision* (%)	*Recall* (%)	*F1* (%)
2	94.11	24.62	30.03
3	87.88	41.43	56.31
4	87.80	72.00	79.11
5	70.45	51.67	59.61
6	72.41	56.21	63.28
7	69.73	79.10	74.12
8	58.06	70.58	63.71
Adaptive	92.00	83.63	87.61

### 3.4 The obstacles perception effectively assists target selection and location

**Figure 8:**
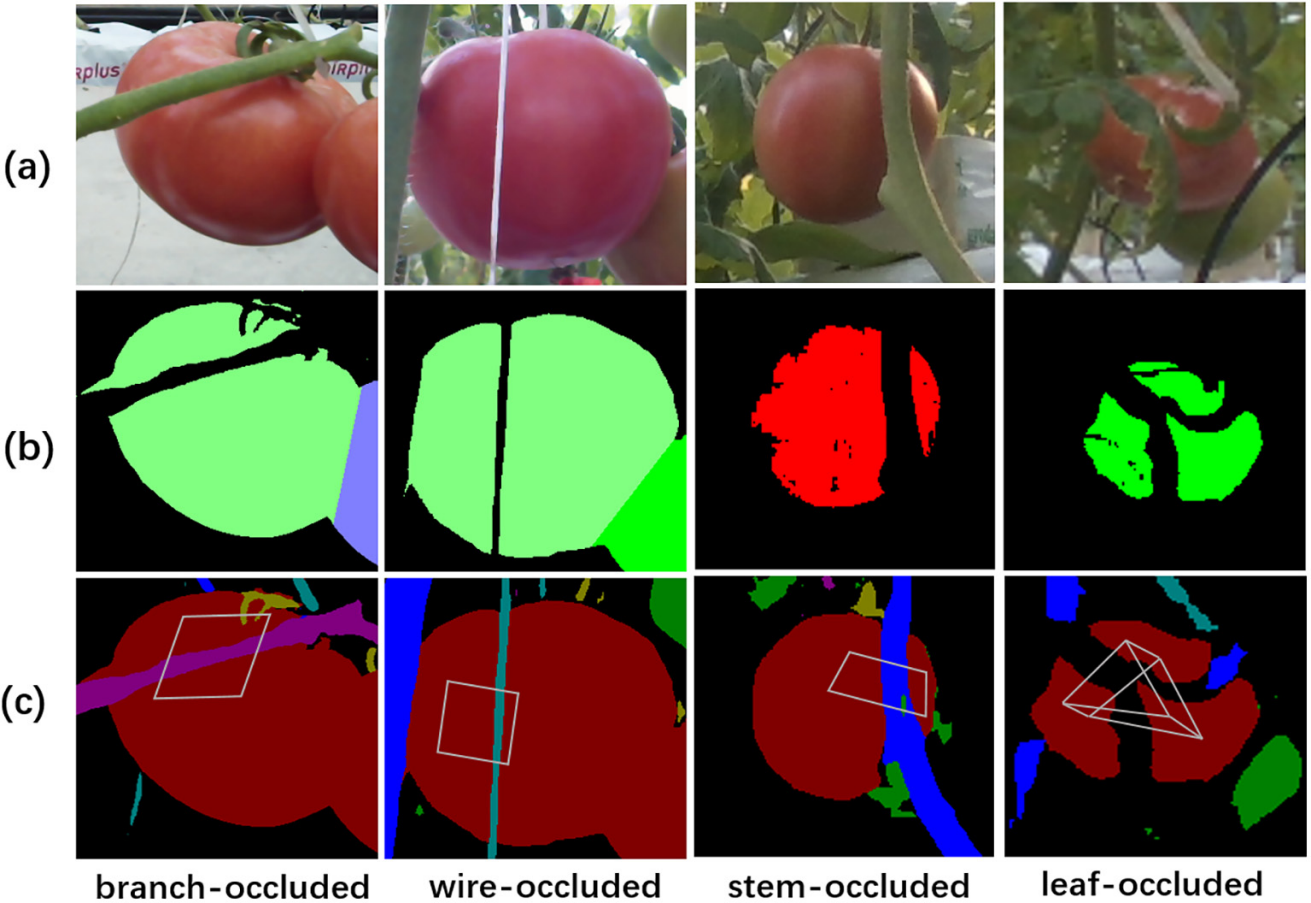
The judgment of the categories of obstructions on occluded tomatoes. **(A)** original RGB images, **(B)** the instances of tomato fruits, **(C)** the semantic segmentation maps and blind spots enclosed by gray quadrilaterals.

The target fruits were selected or discarded according to the judgment of the categories of the obstacles (**Figure 8**). The fruit was discarded if the obstacle was a stem or wire that would hinder the operation of the robotic arm (the first to third columns of **Figure 8**), whereas the fruit was retained if the obstacle to the fruit was a leaf (the last column of **Figure 8**).

To further validate the performance of our method in obstacle perception, we conducted an analysis on all occluded fruits in the validation and testing set (**Table 6**, **Supplementary Material 3**). The results showed that 86.67% of the occluded fruits were successfully perceived, with 96.15% of the obstacle categories were correctly recognized. Specifically, for different types of obstacles including stems, wires, branches and petioles, and leaves, the obstacle detection accuracies were 85.71%, 66.67%, 100%, and 100%, respectively (**Table 6**, **Supplementary Material 3**). The corresponding false detection rates for these obstacle types were 8.33%, 0%, 0%, and 0%, respectively. For different densities of obstacles, such as single obstacle and multiple obstacles, the obstacle detection accuracies were 84.62% and 100%, while the false detection rates were 4.55% and 75%. This indicated that in low-density obstacles environments, there was an increased likelihood of missing fine obstacles such as wires; however, the accuracy of detecting the obstacles categories was high at 95.45%. Conversely, in high-density obstacles scenarios, the obstacles detection accuracy was high at 100%, while accurately identifying all categories of obstacles proved to be more challenging. Additionally, our method achieved an accuracy rate of 86.67% in distinguishing between pickable (obscured by leaves) and non-pickable (obscured by stems, wires or branches) fruits. Specifically, for different types of obstacles, including stems, wires, branches and petioles, and leaves, the judgment accuracies of fruit pickability were 85.71%, 66.67%, 100%, and 100%, respectively (**Table 6**, **Supplementary Material 3**). For different densities of obstacles, such as single obstacle and multiple obstacles, the judgment accuracies of fruit pickability were 84.64% and 100%. This indicated that the accuracy of determining whether the fruit can be picked or not was higher for high-density obstacles compared to low-density obstacles. The above results demonstrated the effectiveness of our approach in recognizing obstructed fruits and preventing potential issues such as mechanical arm entanglement.

**Table 6:** The accuracy of obstacle perception for occluded fruits.

	Categories of obstructions						
	Stems	Wires	Branches and petioles	Leaves	Single obstacle	Multiple obstacles	All fruits
**The perception accuracy of the presence of obstacles (%)**	85.71	66.67	100.00	100.00	84.62	100.00	86.67
**The recognition accuracy of obstruction categories (%)**	91.67	100.0	100.00	100.00	95.45	25.00	96.15
**The judgment accuracy of fruit pickability (%)**	85.71	66.67	100.00	100.00	84.64	100.00	86.67

**Figure 9:**
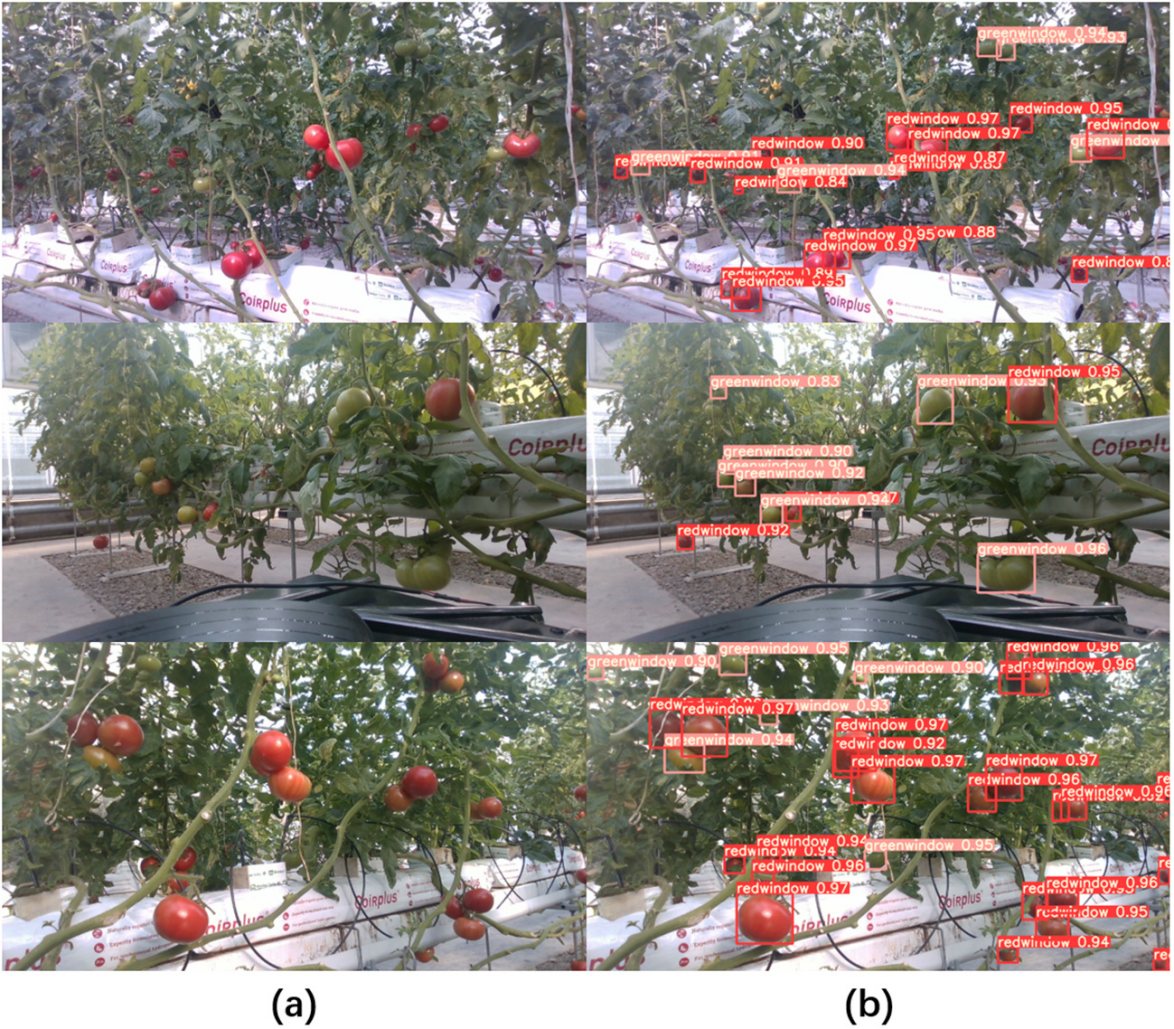
Visualization of the detection results of Yolo v5. **(A)** Original RGB images, **(B)** the detection results, where red window represented mature fruit, green window represented immature fruit and the number represented confidence score.

**Figure 10:**
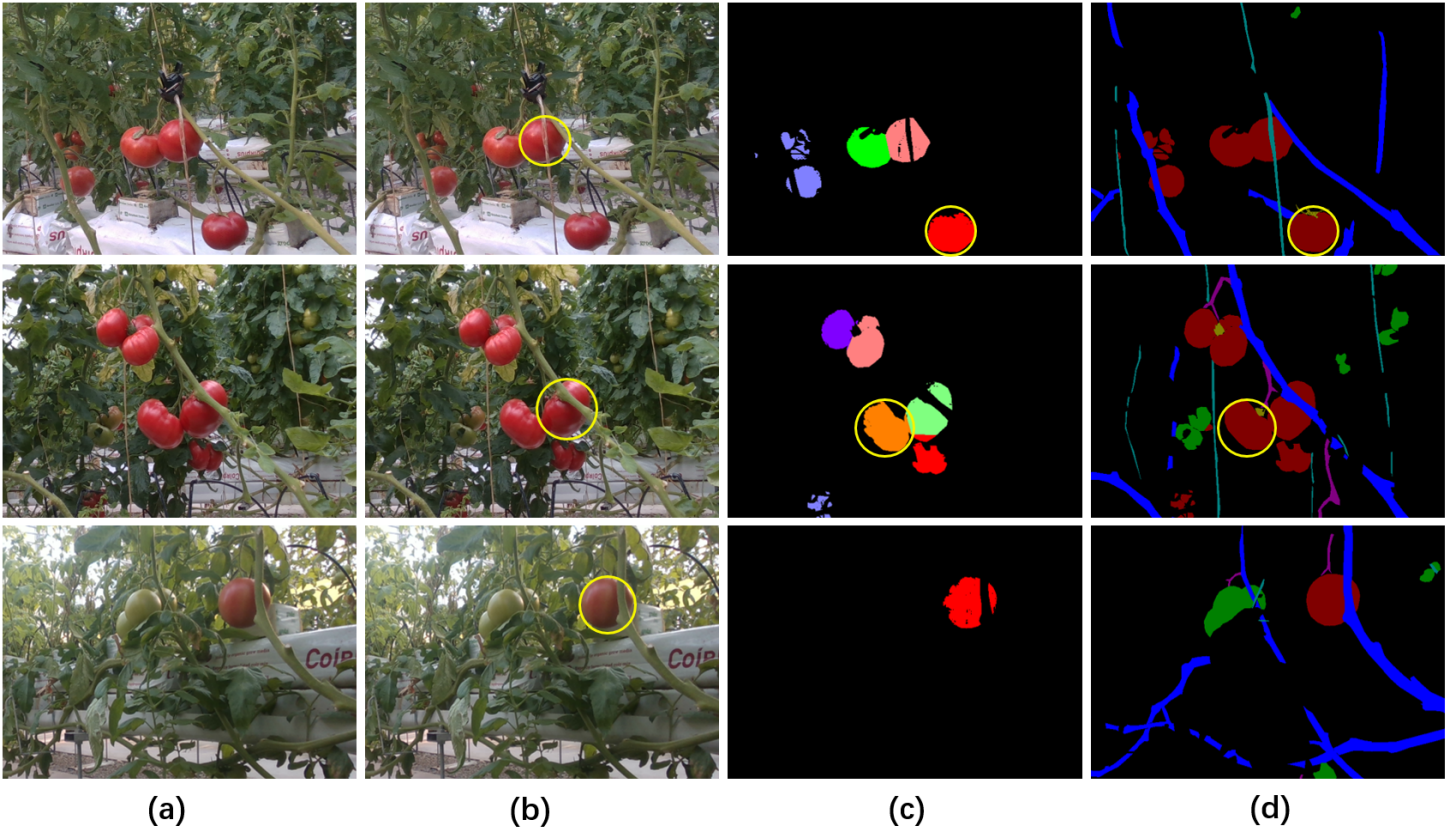
Visualization of the selection results of Yolov5-based method and our method. **(A)** Original RGB images, **(B, C)** the outcomes of Yolov5-based method and our method, respectively, **(D)** manual annotations of the targets in the ground truth images. All detected fruits and annotations were circled in yellow.

The proposed method was compared with the fruit selection and localization methods based on Yolo v5 model (Redmon et al., 2016; Tong et al., 2017) to verify its ability of visual obstacle avoidance. Yolo v5 was employed to detect mature and harvestable fruits. By incorporating depth information, the closest and largest fruit was selected as the target for picking, with its center designated as the picking point. Yolo v5 demonstrated high detection performance for mature fruits in our dataset, achieving a *Precision* of 96.4% and a *Recall* of 92.2%. Although the fruit detection efficiency of Yolo v5 was commendable, numerous occluded fruits were observed in its detection results (**Figure 9**), which would also be selected as picking targets. **Figure 10** showed some selection results of Yolov5-based method and our method, where the targets detected by Yolov5-based method (the fruit selection and localization methods based on Yolo v5) were obstructed by stems or wires (**Figure 10B**), whereas our method can successfully avoid these occluded fruits (**Figure 10C**).

The *Precision*, *Recall* and *F1* of our method in picking targets selection and location were 88.9%, 84.2% and 86.5% (**Table 7**), which were 18.9%, 10.5% and 14.7% higher than that of Yolov5-based method. The better performance of our method was largely attributed to the fact that our method can remove the occluded fruits. The mean *PE* between the detected results and the annotations of picking targets were calculated, and the mean *PE* of our method and that of Yolov5-based method were compared (**Table 7**). The mean *PE* of our method was 9.5% with an average radius of 48.85 pixels, which was obviously superior than that of Yolov5-based method, with a proportion exceeding 10%, partly because our method used the minimum circumcircle of the visible and blind plots of the fruit as the picking target, which reduced the center point offset when part of the fruit was missing due to the occlusion of obstacles.

**Table 7:** Quantitative evaluation of the performance of two methods in picking-target selection and location.

Method	*Precision* (%)	*Recall* (%)	*F1* (%)	mean *PE* (%)
**Yolov5-based method**	70.0	73.7	71.8	10.8
**Our method**	88.9	84.2	86.5	9.5

**Figure 11:**
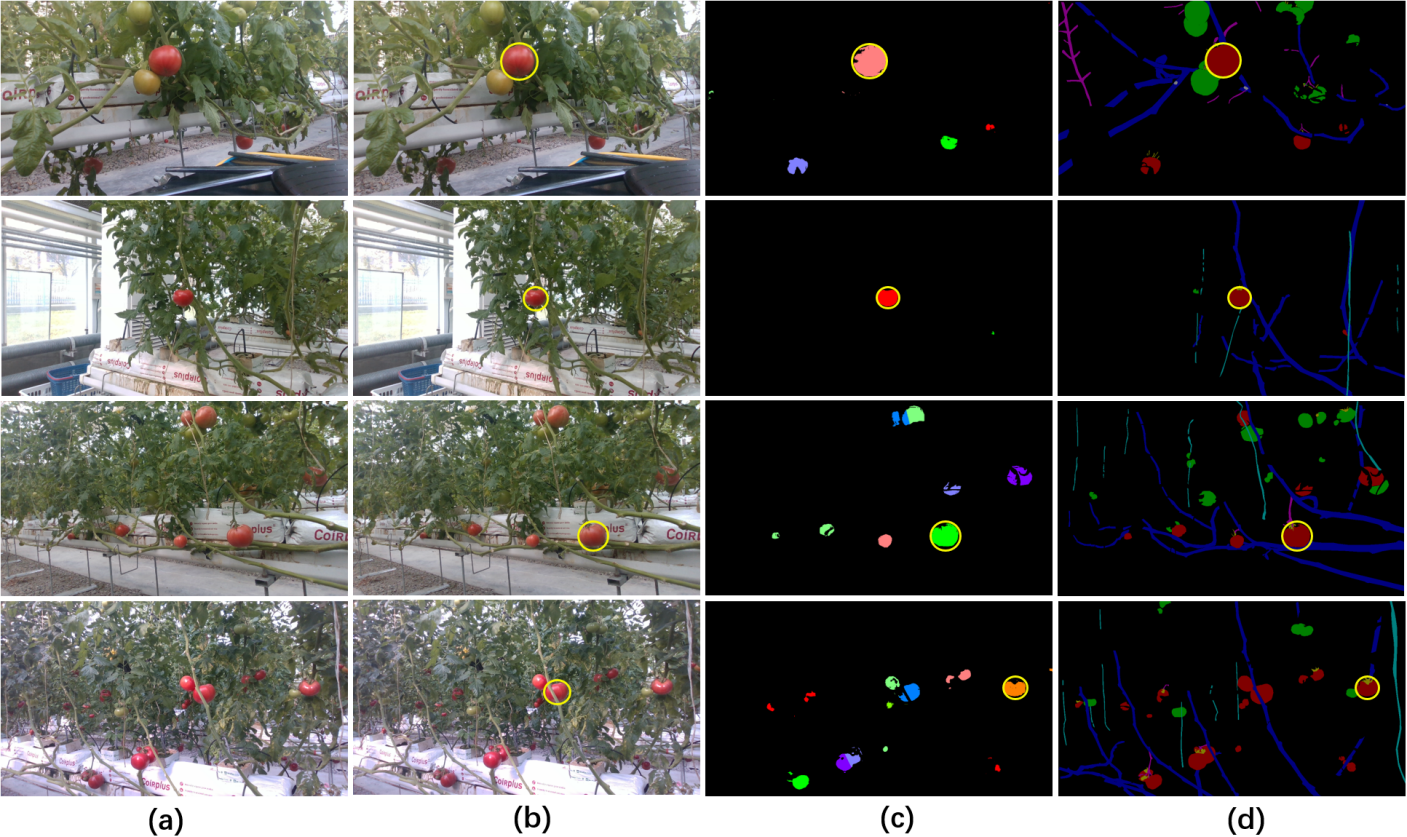
Visualization of the selection results of Yolov5-based method and our method under different obstacle complexities and densities. The two upper rows illustrated results under sparse obstacles, while the two lower rows illustrated results under dense obstacles. **(A)** Original RGB images, **(B)** and **(C)** the outcomes of Yolov5-based method and our method, respectively, **(D)** manual annotations of the targets in the ground truth images. All selected fruits and annotations were circled in yellow.

Additionally, we categorized the images into two groups (one group comprised 26 images featuring dense obstacles, whereas the other included 24 images characterized by sparse obstacles) to evaluate the performance of our method under varying obstacle complexities and densities. **Figure 11** illustrated the selection results of both Yolov5-based method and our proposed method across varying complexities and densities of obstacles. The visualization results indicated that both methods effectively selected the correct target in scenarios with low obstacle complexity and density, however, as occlusion intensified, Yolov5-based method faced increased challenges in avoiding occluded fruits. The quantitative comparison results indicated that our proposed method (precision=87.5%) significantly outperformed Yolov5-based method (precision=58.3%) in scenarios with dense obstacles (**Table 8**), largely due to its effective obstacle detection capabilities. In scenarios with fewer obstacles (**Table 9**), both our method and Yolov5-based method exhibited commendable fruit selection performance, achieving precision of 90.9% and 90.4%, respectively. Our method demonstrated effective fruit selection and localization performance across scenes with varying obstacle complexity and density, thereby validating its robustness and applicability.

**Table 8:** The quantitative comparison of our method and Yolov5-based method on images with dense obstacles.

Method	*Precision* (%)	*Recall* (%)	*F1* (%)
**Yolov5-based method**	58.3	63.6	60.8
**Our method**	87.5	80.8	84.0

**Table 9:** Comparison results of our method and Yolov5-based method on images with sparse obstacles.

Method	*Precision* (%)	*Recall* (%)	*F1* (%)
**Yolov5-based method**	90.4	86.3	88.3
**Our method**	90.9	86.9	88.9

### 3.5 Computational efficiency analysis

On the images with a resolution of 600×800 pixels, the average time consuming for each phase of our method was 0.018s, 0.8s, and 0.013s, respectively, and the total time consuming was less than 1s. We also investigated the time consumption of the same input on Yolov5-based method. Both methods were implemented based on Python programming language and tested on single NVIDIA GeForce GTX1660. Although our method took about 0.6 seconds longer than Yolov5-based method, the total time was still very short and acceptable for current harvesting robots. This can be understood as sacrificing a reasonable time to perceive obstacles within the fruits.

## 4 Discussion

Robotic harvesting is a complex task that integrates multidisciplinary expertise, including kinematics, control systems, machine vision, and behavioral science. A pivotal initial step for harvesting robots is to accurately perceive the target fruit and its surrounding environment through advanced machine vision techniques. To enhance the efficiency of the picking process, it is imperative that the vision system of harvesting robots delivers comprehensive information, encompassing fruit recognition, ripeness assessment, 3D coordinate localization, obstacle detection, and estimation of the fruit’s 3D posture, and so on. Objective detection algorithms can effectively distinguish ripe and unripe fruits, and by integrating depth image information, the 3D coordinates of the fruits can be accurately located. While, obstacle recognition has always been a challenge and is often overlooked in the vision systems of harvesting robots. Accurate obstacle recognition is crucial for determining harvestable targets and planning efficient harvesting paths.

To address the issue of fruit occlusion during picking, a prioritized selection method for unobstructed fruits was proposed in this study. The proposed method achieved target recognition and localization while also perceiving obstacles, demonstrating its effectiveness in picking target selection and location in intricate scenarios. However, revisions were still necessary to further enhance the capacity of the method. The accuracy of target selection and location largely depends on the performance of the image semantic segmentation and the pixel clustering for instance differentiation. The segmentation of obstacles such as stems basically meet the requirement of the target selection algorithm, but the segmentation accuracy of extremely slender obstacles such as wires still need to be further improved. Also, two typical issues may arise during the processes of instance differentiation and obstacle perception. The first issue pertains to the unsuccessful separation of tightly overlapping fruits, resulting in their classification as a single harvesting target. To alleviate this problem, it is imperative to enhance pixel clustering by incorporating more comprehensive judgments of the instances for improved segmentation performance, or consider employing deep learning for fruit instance segmentation. The second issue pertains to the neglect of obstacles located on the edge of the fruits by the perception algorithm. Therefore, further optimization of the obstacle perception algorithm was essential, such as augmenting its ability to detect obstacles surrounding fruit edges, or avoiding this potential risk by providing more fruit pose information to coordinate with fruit grasping actions.

Our proposed method demonstrated an appreciable ability in selecting and locating barrier-free mature fruits within non-structural environments, especially avoiding fruits obscured by stems or wires. This advancement offers a more reliable and practical solution for fruit recognition and localization of harvesting robots. However, there are still certain limitations, such as the absence of detailed information regarding the posture of fruit. Accurate fruit pose estimation is crucial for identifying optimal grasping points and planning the most efficient harvesting path and actions. Achieving 3D pose estimation of tomatoes within complex growth environments, while synchronizing it with the posture of the end-effector, is essential for minimizing damage to surrounding fruits and foliage, significantly improving the success rate of harvesting. In the future, we intend to estimate the posture of fruits through the integration of 2D and 3D visual features in conjunction with keypoint detection, serving the motion path planning of the robotic arm and guiding the grasping posture of the gripper, thereby enabling high-efficiency and higher-quality fruit picking. Additionally, the vision system must be capable of addressing various weather conditions and changes in lighting, as well as adapting in real-time to dynamic changes such as the movement of fruits or foliage, ensuring sustained accuracy in fruit recognition and positioning. Consequently, significant efforts are still required to enhance the robustness and real-time performance of the algorithms.

## 5 Conclusions

Encountering obstacles during the mechanical picking process of tomatoes is inevitable, despite the growing environments are gradually transitioning from unstructured to semi-structured. These obstacles, such as slender stems obstructing the fruits, often entangled with robotic arms and result in picking failures. Our research introduced a novel method for selecting and locating barrier-free fruits based on semantic segmentation and obstacle perception algorithm that offers a solution for selecting safe targets for harvesting robots in practical applications. Each phase of the proposed method offers distinct advantages. Firstly, the easy-to-use spatial-relationship feature module designed for the image semantic segmentation enabled finer segmentation of slender structural objects by discovering the spatial relevance with features. Secondly, incorporating depth maps as input of adaptive K-means clustering significantly enhanced the performance of fruit instance segmentation. Lastly, our proposed obstacle perception and target selection algorithm can effectively select and discard both non-occluded and occluded fruits. Experiments conducted on our Tomato Dataset and our Tomato Harvesting Robot Platform demonstrated that the proposed spatial-relationship features greatly improve semantic segmentation performance while showcasing our method’s ability to exclude obstructed fruits in target selection and location. It is worth noting that our proposed method possesses the potential for extension to a wide range of similar target recognition and localization tasks related to commonly cultivated fruits and vegetables, such as peppers, apples, and kiwis, demonstrating universality and versatility applicability. The model’s generalization ability suggests that it can be calibrated and deployed across various agricultural scenarios and scales, thereby providing a robust solution for automated detection systems within the smart farming sector.

The current methodology, while promising, still faces several challenges in the visual task for robotic fruit harvesting. Firstly, there exists a deficiency in the provision of comprehensive posture information regarding fruits and their stems, which is essential for achieving precise robotic manipulation. Additionally, the segmentation accuracy for extremely slender obstacles, such as wires, necessitates further enhancement. The algorithm also faces difficulties in effectively distinguishing and segregating fruits that are heavily overlapping or entangled. Furthermore, to effectively perceive the dynamic changes of fruits, branches, and other obstacles during the harvesting process, there is a pressing need to enhance both the computational efficiency and real-time performance of the algorithm.

In our future research, we intend to construct an integrated end-to-end network informed by the concepts presented in this paper. Multi-task learning networks present a promising approach to achieving this objective, as they effectively balance efficiency while addressing multiple parsing tasks within the visual perception system of fruit harvesting robots. Furthermore, we will refine the backbone of the network to enhance its ability for segmenting extremely slender obstacles, such as wires. Concurrently, we will utilize the powerful feature extraction capabilities of the neural network to enhance the performance of fruit instance segmentation. Additionally, in the new design, we also plan to add the fruit pose estimation through the integration of 2D and 3D visual features in conjunction with keypoint detection. This capability is crucial for guiding robotic grippers to approach and grasp the fruits from the most advantageous angles, thereby minimizing the risk of damage. This integrated network will not only facilitate accurate fruit selection and positioning, but also support motion path planning for robotic arms and guide gripping postures during fruit harvesting. These enhancements will yield more comprehensive, accurate and efficient visual information, while enhancing the harvesting robot’s ability to adapt to the unique posture of each fruit and to avoid complex obstacles surrounding it. This is invaluable for further improve the success rate and efficiency of fruit harvesting in unstructured complex environments.

## Data Availability

The original contributions presented in the study are included in the article/**Supplementary Material**. Further inquiries can be directed to the corresponding authors.
