# Supplementary material for: Barrier-free tomato fruit selection and location based on optimized semantic segmentation and obstacle perception algorithm
Source: Front Plant Sci. 2024 Oct 31;15:1460060. doi: 10.3389/fpls.2024.1460060 (PMC11560766; doi:10.3389/fpls.2024.1460060)
Supplement: Supplementary file 1 [file DataSheet1.docx]

Supplementary Material

# The algorithm flowchart of obstacle perception


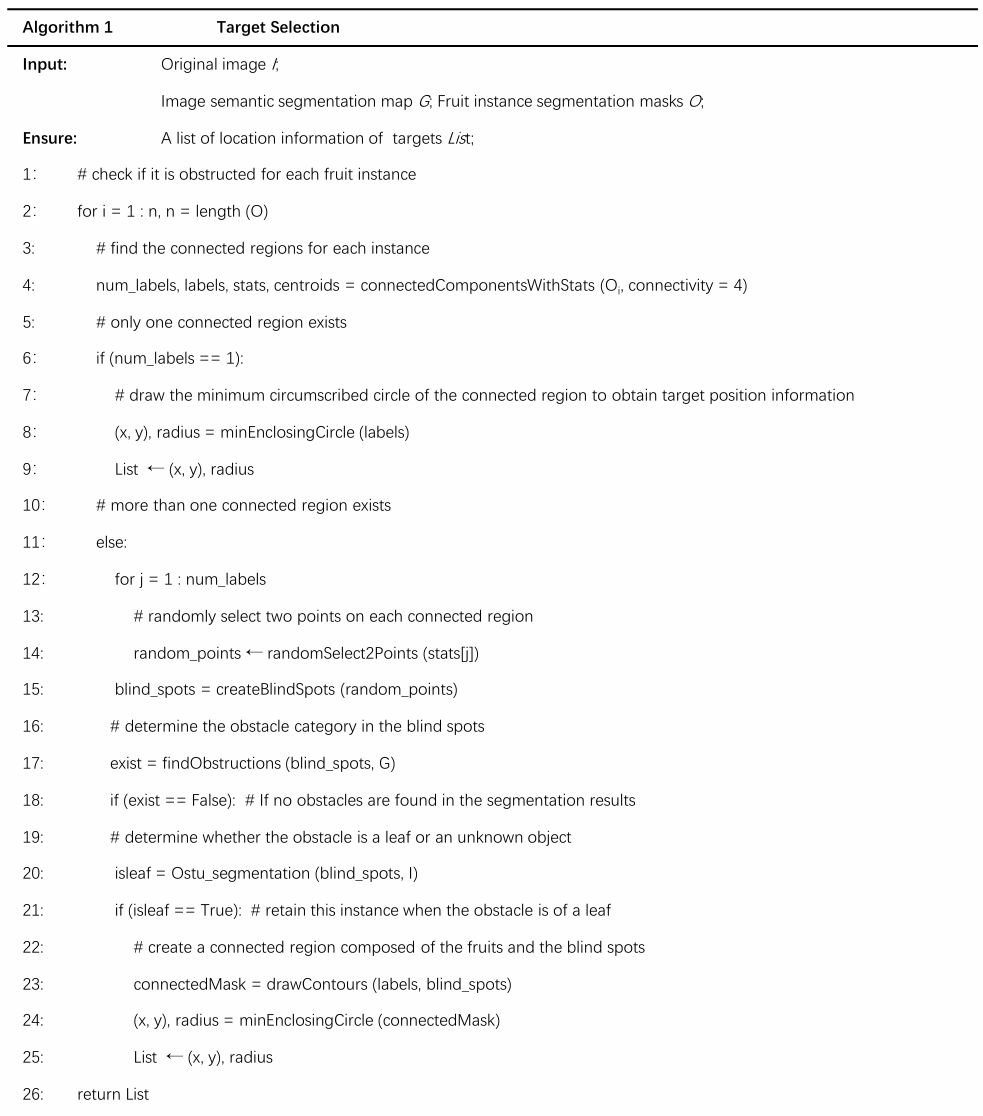


**Supplementary Figure 1.** The algorithm flowchart of obstacle perception

# Improvement of semantic segmentation performance on a Pepper dataset

A synthetic Pepper dataset produced by Barth et al. (Barth et al., 2018) was also used to obtain feedback for modelling improvements. The dataset contains 10500 images with a resolution of 600×800 pixels and corresponding semantic segmentation annotations. Barth et al. fully demonstrated the high similarity between synthetic images and empirical images, and further verified the availability of synthetic bootstrapping and empirical fine-tuning (Barth et al., 2018). We randomly selected 3000 synthetic images as training data, 50 synthetic images as validation data and 50 synthetic images as testing data. The acc and mIoU results were shown in Supplementary Table 1, both of the values had a certain improvement when SFM was added to the models. Thanks to SFM's ability to extract spatial features, the IoU values of stems and wires have been significantly improved (Supplementary Table 2), which can be more intuitively seen in Supplementary Figure 2.

**Supplementary Table 1 Comparison between baselines and networks with SFM of *acc* and *mIoU* on the Pepper testing set.**

| **Method** | ***acc*(%)** | ***mIoU*(%)** |
| --- | --- | --- |
| U-net w/o SFM on the Pepper testing set | 88.36 | 49.13 |
| U-net w/ SFM on the Pepper testing set | 89.58 | 50.12 |
| DeepLab v3 w/o SFM on the Pepper testing set | 89.55 | 53.84 |
| DeepLab v3 w/ SFM on the Pepper testing set | 90.56 | 58.77 |

**Supplementary Table 2 *IoU* of each class on the Pepper testing set.**

| **Method** | **Background (%)** | **Leaves**  **(%)** | **Pepper (%)** | **Stems (%)** | **Shoots and leaf stems (%)** | **Wires (%)** |
| --- | --- | --- | --- | --- | --- | --- |
| U-net w/o SFM | 83.11 | 84.81 | 80.08 | 41.72 | 40.61 | 26.65 |
| U-net w/ SFM | 83.50 | 86.96 | 80.17 | 43.04 | 42.20 | 28.57 |
| DeepLab v3 w/o SFM | 76.73 | 81.54 | 84.30 | 52.50 | 42.51 | 24.28 |
| DeepLab v3 w/ SFM | 76.35 | 82.45 | 83.46 | 62.24 | 47.00 | 35.75 |


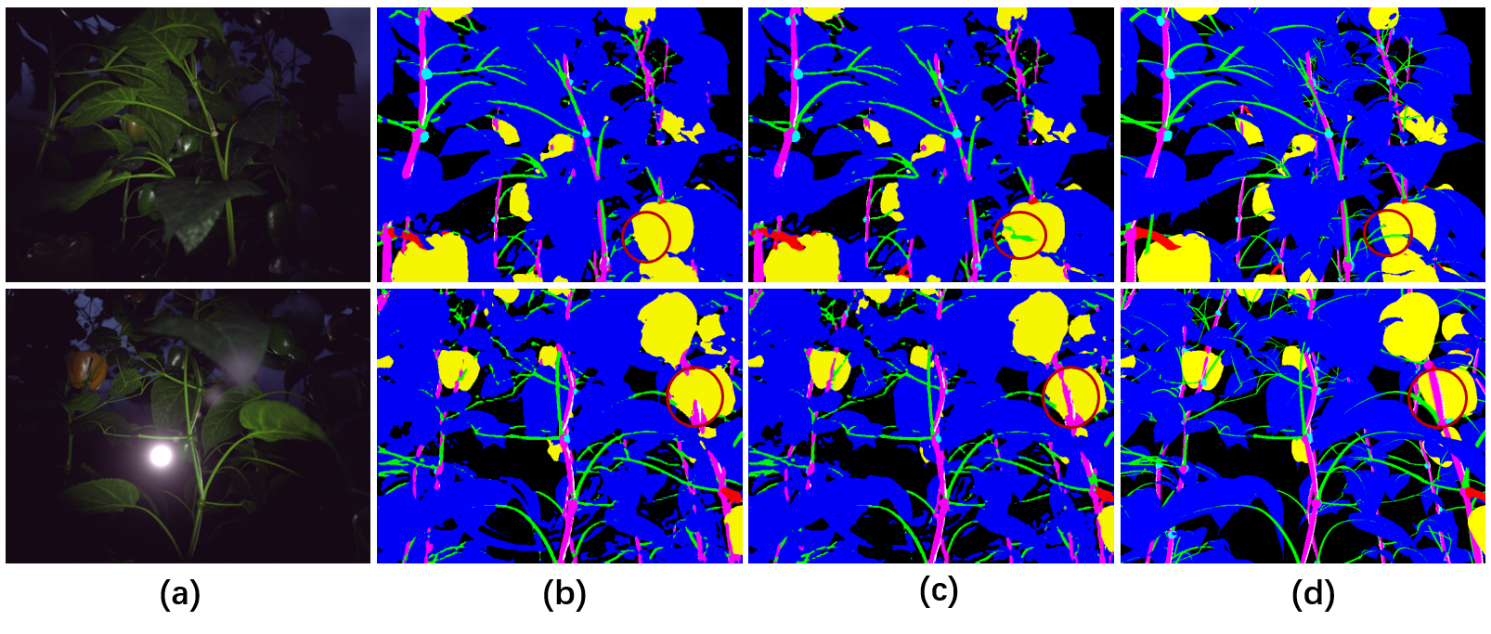


**Supplementary Figure 2.** Visual comparison of the parsing results of DeepLab v3 and DeepLab v3 with SFM on the Pepper testing set. Class labels: black indicates background, dark blue indicates leaves, yellow indicates peppers, red indicates peduncles, purple indicates stems, green indicates shoots and leaf stems, light blue indicates wires and grey indicates cuts. (a) Original images, (b) the segmentation results of DeepLab v3, (c) the segmentation results of DeepLab v3 with SFM, (d) the ground truth labels.

# The detailed perception results of all occluded fruits

Supplementary Table 3 The detailed perception results of all occluded fruits

| **No.** | **Category of obstruction** | **Category of detected obstruction** | **Manual judgment** | **Judgment of our method** |
| --- | --- | --- | --- | --- |
| 1 | B | B | ⅹ | ⅹ |
| 2 | L | L | √ | √ |
| 3 | W | W | ⅹ | ⅹ |
| 4 | S | S | ⅹ | ⅹ |
| 5 | S | S | ⅹ | ⅹ |
| 6 | W | W | ⅹ | ⅹ |
| 7 | W | N | ⅹ | √ |
| 8 | W and S | S | × | × |
| 9 | W | N | × | √ |
| 10 | S | W | × | × |
| 11 | B | B | × | × |
| 12 | S | S | × | × |
| 13 | S | S | × | × |
| 14 | S | S | × | × |
| 15 | W | W | × | × |
| 16 | L | L | √ | √ |
| 17 | S | S | × | × |
| 18 | B | B | × | × |
| 19 | S | S | × | × |
| 20 | S | N | × | √ |
| 21 | W and S | S | × | × |
| 22 | S | S | × | × |
| 23 | W and S | W and S | × | × |
| 24 | S | S | × | × |
| 25 | S | S | × | × |
| 26 | L | L | √ | √ |
| 27 | S | S | × | × |
| 28 | W | W | × | × |
| 29 | S | N | × | √ |
| 30 | W, S and L | W and S | × | × |

**^a^ B denotes branches and petioles, S denotes stems, W denotes wires, L denotes leaves, N denotes no obstruction,** √ **indicates the fruit can be picked, × indicates the fruit can not be picked**

# References

Barth, R., IJsselmuiden, J., Hemming, J. and Van Henten, E.J., 2018. Data synthesis methods for semantic segmentation in agriculture: A Capsicum annuum dataset. Computers and electronics in agriculture, 144, pp.284-296. doi: 10.1016/j.compag.2017.12.001
